# Effect of a trub, hops, and yeast mixture on jejunal mucosal immune status, oxidative stress status, structure, and growth performance of nursery pigs

**DOI:** 10.1093/jas/skaf425

**Published:** 2025-12-10

**Authors:** Alexa R Gormley, Zixiao Deng, Brock Ashburn, Robert W Bryant, Sung Woo Kim

**Affiliations:** Department of Animal Science, North Carolina State University, Raleigh, NC, 27695; Department of Animal Science, North Carolina State University, Raleigh, NC, 27695; Highland Brewing Company, Asheville, NC, 28803; Department of Chemistry and Physics, Warren Wilson College, Swannanoa, NC, 28778; Department of Animal Science, North Carolina State University, Raleigh, NC, 27695

**Keywords:** hops, immune response, intestinal health, nursery pigs, oxidative stress, yeast

## Abstract

The craft brewing process results in the generation of co-products that have potential value in replacing traditional feedstuffs. A trub, hops, and yeast mixture (THYM) derived from the craft brewing process contains high levels of hop acids and yeast cells. The purpose of this study was to investigate the effects of increasing levels of THYM in diets of nursery pigs on the jejunal mucosal immune and oxidative stress status, morphological parameters, and growth performance. Thirty-two pigs (6.8 ± 0.3 kg body weight) weaned at 3-wk-of-age were allotted into 4 dietary treatments, using a randomized complete block design, with sex and initial body weight as blocks. The dietary treatment consisted of the basal diet supplemented with 0.0, 0.7, 1.4, or 2.1% THYM. The THYM replaced a mixture of 40% corn and 60% soybean meal in the basal diets. Pigs were fed for 28 d in 3 phases (9, 11, and 8 d, respectively). On d 28, all pigs were euthanized for sampling of jejunal tissue and mucosa to analyze immune and oxidative stress status. The increasing levels of THYM linearly increased (*P *< 0.05) gain to feed ratio of pigs, however, average daily gain and feed intake were not affected. Increasing levels of THYM linearly increased (*P *< 0.05) the relative gene expression of nucleotide oligomerization domain 2 and interferon-γ and tended to increase (*P = *0.058) relative gene expression of Toll-like receptor 2 in the jejunum. Increasing levels of THYM did not affect the amount of immunoglobulins or oxidative damage products in the jejunal mucosa. The increasing levels of THYM linearly increased (*P *< 0.05) the percentage of Ki-67^+^ cells in the crypt, whereas villus height and crypt depth were not affected. Overall, the inclusion of the trub, hops, and yeast mixture obtained from the craft brewing process may have contributed to the increased rate of intestinal tissue repair following weaning stress, potentially explaining the improved gain-to-feed ratio in nursery pigs. Furthermore, the increasing inclusion of THYM increased the expression of genes related to innate immune system recognition and signaling without causing the elevation of inflammatory responses and humoral immune responses, or the associated oxidative stress.

## Teaser

The use of a trub, hops, and yeast mixture derived from the craft brewing process improved feed efficiency, increased expression of genes associated with innate immune system recognition and signaling, and increased proliferation of stem cells in the small intestine when included at up to 2.1% of the diets for nursery pigs.

## Introduction

The weaning period is widely considered to be the most stressful period in the life of a pig (Hu et al., 2013). At this stage, the pig’s intestine is still considered immature and weaning leads to disruption of the mucosa-associated microbiota (Moita and Kim, 2023; Deng et al., 2023a), increased intestinal inflammation (Moeser et al., 2017; Duarte et al., 2023), decreased efficiency of nutrient absorption (Yuan et al., 2017; Deng et al., 2022), and increased susceptibility to disease (Fabà et al., 2020). The jejunum is the major site for digestion and absorption of amino acids, monosaccharides, and fatty acids (Lewis and Southern, 2000) and is also where significant disruption to intestinal barrier and immune functions can occur due to anti-nutritional and harmful compounds from the feed (Kim and Duarte, 2021). Nutritional interventions can be utilized to support intestinal health and mitigate the negative impacts of weaning. These effects can include positive modulation of the mucosa-associated microbiota (Moita et al., 2021; Baker et al., 2024a), activation of intestinal pattern recognition receptors (Duarte and Kim, 2024; Gormley et al., 2024), increased antioxidant and anti-inflammatory activity (Li et al., 2013; Pearce et al., 2024; Garavito-Duarte et al., 2025a), and enhancement of intestinal morphology and barrier function (Duarte et al., 2024; Garavito-Duarte et al., 2025b). These effects may contribute to improved nutrient digestion and utilization (Almeida et al., 2013; Chen et al., 2020). Nutritional interventions that are designed to support intestinal health can impose an increased financial burden on pig production and thus the value creation of co-products derived from other industries would be beneficial to pig production (Woyengo et al., 2014; Kim et al., 2024).

Since 1994, the number of craft breweries in the United States has increased from less than 600 to almost 10,000 in 2024 (Brewers Association, 2025). Brewers grain is a common co-product that has been effectively used as feedstuffs for ruminants (Preston et al., 1973; Faccenda et al., 2019) but is not as applicable for non-ruminants due to its relatively high fiber content and poor protein quality. Other downstream co-products of the craft brewing process, like trub, hops, and yeast, may have potential for use in feeding to non-ruminant animals. Trub is a high protein sediment that is obtained after the “hot-side brewing” process that is approximately 50% crude protein on a dry matter basis (Mathias et al., 2015). Hops, added to give beer its flavor and aroma (Korpelainen and Pietiläinen, 2021), produce hop acids and metabolites, which are known to have antimicrobial and antioxidative properties (Zanoli and Zavatti, 2008; Kolenc et al., 2022). Spent yeast, another high protein co-product obtained after fermentation, consists of yeast cells along with residual hop acids and metabolites, yeast metabolites and cell wall fragments. The use of trub as a feed ingredient for animals has garnered limited research attention. Hop acids and metabolites have been previously investigated in the diets of broiler chickens and pigs for their antimicrobial and antioxidative properties (Bortoluzzi et al., 2014; Sbardella et al., 2016; Ząbek et al., 2020). Yeast-derived compounds have also been previously studied and are known to positively modulate the jejunal mucosa-associated microbiota, detoxify toxic compounds, and stimulate immunocompetence (Shen et al., 2009; Deng et al., 2023a; Gormley et al., 2024). Considering the documented effects of hop acids and metabolites and yeast-derived compounds, along with the unexplored potential of trub, using these co-products in combination may elicit synergistic benefits to intestinal health through their combined influences on the mucosa-associated microbiota and the intestinal immune response. This warrants investigation for their combined use in the diets of nursery pigs.

A trub, hops, and yeast mixture derived from the craft brewing process may serve as an effective nutritional intervention to support intestinal health in nursery pigs and provide a sustainable, value-added purpose for these co-products. Therefore, it was hypothesized that a co-product from craft brewing process including trub, hops, and yeast could positively influence the mucosa-associated microbiota, immune response, oxidative stress status, and villus structure and repair in the jejunum, thereby impacting growth performance of pigs. The objective of this study was to investigate the effects of increasing levels of a trub, hops, and yeast mixture in the diets on the mucosa-associated microbiota, immune and oxidative stress status, villus structure and repair in the jejunum, and growth performance of nursery pigs.

## Materials and Methods

The procedure of this study was reviewed and approved by the North Carolina State University Institutional Animal Care and Use Committee (Raleigh, NC, USA). This experiment was conducted at the Metabolism Education Unit of North Carolina State University (Raleigh, NC, USA). The trub, hops, and yeast mixture (THYM) was obtained from Highland Brewing Company (Asheville, NC, USA).

### Preparation and evaluation of the trub, hops, and yeast mixture

The trub, hops, and yeast mixture was obtained in a slurry form, containing 8% solids, from Highland Brewing Company (Asheville, NC, USA) as a co-product of the craft brewing process. Hot trub was collected in the whirlpool process after the initial grain mashing, lautering, and boiling steps. Flocculated spent yeast was collected from chilled fermenter tanks at the conclusion of the fermentation process. Spent yeast in craft brewing is rich in hops and particularly so in dry hopped beer. The combination of the hot trub and the spent yeast contains the specific components of trub, hops, and yeast. To dry THYM, the THYM in liquid form was delivered to Valentine Chemical (Lockport, LA, USA) for drum-drying (Almena et al., 2019). The liquid was homogenized before being applied to the exterior of a drum filled with 200°C steam. The dried THYM was then scraped off the drum within 3 to 5 s and was subsequently packaged in mylar bags. The approximate composition of THYM was calculated using measured hop acid and metabolite contents, values obtained from nutrient analysis, and reference values of spent yeast and trub (Table 1). The total hop acid contents in each diet were calculated by multiplying the hop acid content of THYM by the THYM inclusion in the diet and were expressed on a mg/kg basis (Table 2).

**Table 1.** Composition of a trub, hops, and yeast mixture (THYM; as-fed basis)^1^

Item	THYM
**Dry matter, %**	93.7
**Metabolizable energy, kcal/kg**	3,920
**Crude protein, %**	33.9
**Standardized ileal digestible Lys, %**	1.65
**Calcium, %**	0.53
**Phosphorous, %**	2.02
**Alpha acids^2^, mg/g**	19.4
**Beta acids, mg/g**	17.0
**Iso-alpha acids, mg/g**	86.4
**Humulinones, mg/g**	1.20
**Xanthohumol, mg/g**	3.10

1 Comprehensive assessment of hop acids and metabolite contents were conducted by HopTechnic (Virgil Gamache Farms, Inc., Toppenish, WA, USA).

**Table 2.** Composition of hop acid and metabolite content in the experimental diets (as-fed basis)

	THYM^1^, %			
Item, mg/kg	0.0	0.7	1.4	2.1
**Alpha acids**	<1	135	271	406
**Beta acids**	<1	119	238	357
**Iso-alpha acids**	<1	604	1,209	1,813
**Humulinones**	<1	8	17	25
**Xanthohumol**	<1	22	43	65

1 Trub, hops, and yeast mixture derived from craft brewing process, drum-dried.

### Quantification of yeast cell lysis after heat treatment

To determine the effects of drum-drying on yeast cell lysis, drum-drying was simulated. Samples of yeast slurry were obtained from Highland Brewing Company (Ashville, NC, USA) and were divided into two categories, with and without heat treatment. For samples subjected to heat treatment, an aluminum pan was heated using a hot plate until the surface reached 125°C. Temperatures were verified using a thermal imaging camera (FLIR C5, Teledyne FLIR, Wilsonville, OR, USA) before spreading a 2 mm thick layer of yeast slurry and allowing the slurry to dry for 1 min before removing from the heat source. The dried yeast was then scraped from the pan using a sharp blade. For samples not subjected to heat treatment, the samples of yeast slurry were stirred until homogenized and poured into metal sheet pans at a thickness of 3 cm and were frozen for 3 d at –20°C. Using a freeze-dryer (VirTis General Purpose, Scientific Products, Warminster, PA, USA), samples were freeze-dried using a preprogrammed recipe provided by the machine operation manual. The recipe included eight steps where the machine cycled at the following temperatures and hold times, 1) –30°C for 45 min; 2) –20°C for 45 min; 3) –15°C for 45 min; 4) 0°C for 180 min; 5) 10°C for 360 min; 6) 15°C for 600 min; 7) 30°C for 600 min; 8) 40°C for 1,200 min. All temperature cycles were conducted at a pressure of 0.66 Pa and complete drying of the samples took approximately 2 d. After drying, both sample types were ground into a uniform powder using a mortar and pestle.

**Figure 1.**
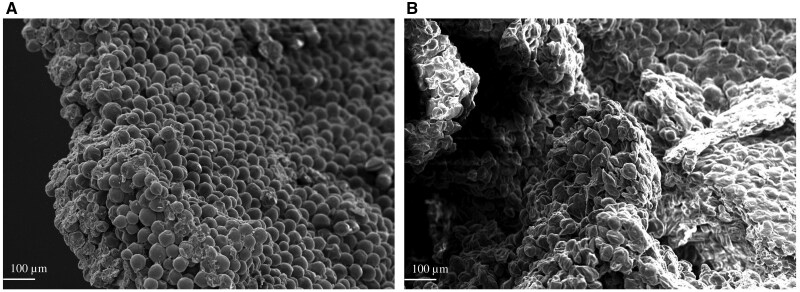
Images of yeast slurry not subjected to heat treatment (A) and yeast slurry subjected to heat treatment (B) taken by Variable Pressure Scanning Electron Microscope (Hitachi SU3900, Hitachi High-Tech, Tokyo, Japan). Samples of yeast slurry not subjected to heat treatments were subjected to freeze drying procedures where the maximum temperature did not exceed 40°C; samples of yeast slurry subjected to heat treatment were spread to approximately 1–2 mm of thickness and dried on a flat, stainless steel surface that was heated to 125°C for 1 min to simulate heat damage associated with drum-drying.

The dried samples were then taken to the Analytical Instrumentation Facility at North Carolina State University (Raleigh, NC, USA), where they were prepared for imaging using scanning electron microscopy. Briefly, samples were mounted onto discs using carbon fiber tape and were coated using argon gas on a sputter coater (208HRD-180 High Resolution Sputter Coater, Cressington Scientific Instruments, Watford, UK). The coated discs were then loaded into a Variable Pressure Scanning Electron Microscope (Hitachi SU3900, Hitachi High-Tech, Tokyo, Japan) and images were taken by a trained technician. The resulting images were then sectioned into equal sized segments and four representative segments from each image were counted for the total number of lysed and intact cells. Lysed cells were considered to be cells that appeared cavitated or fragmented, as described by Gnoinski et al. (2021), who described cells with this altered morphology to be associated with degradation and increased porosity of the cell wall. Intact cells were considered to be yeast cells that appeared to be intact with a smooth appearance of the cell wall. The number of lysed yeast cells and total yeast cells per image segment were used to calculate an average percentage of lysed yeast cells for samples dried with and without heat treatment (Figure 1). Three segmented images were counted per treatment (*n* = 3) and the average yeast cell lysis per treatment was calculated to be 92.6 and 6.8% lysed, for samples dried with and without heat treatment, respectively.

### Experimental design, animals, and diets

Thirty-two newly weaned pigs (PIC 337 × Camborough 22; 16 barrows and 16 gilts) at 6.8 ± 0.3 kg body weight (BW) were weaned at d 21 of age and were allotted into 4 dietary treatments using a randomized complete block design with initial BW (light and heavy) and sex serving as blocks. Each pig was given free access to feed and water and all pigs were housed individually. There were 8 replicates per treatment group. The dietary treatment consisted of the basal diet, supplemented with 0.0, 0.7, 1.4, or 2.1% THYM. Basal diets were formulated to meet or exceed the NRC (2012) requirements. In the experimental diets the THYM replaced a 60% soybean meal, 40% corn mixture in the basal diet. Pigs received their respective treatments for 28 d in 3 phases: phase 1 for 9 d (to 7 kg BW), phase 2 for 11 d (7 to 11 kg BW), and phase 3 for 8 d (11 kg BW to the end of the study). The composition of the experimental basal diets is shown in Table 3. Experimental diets were produced at the Feed Mill Educational Unit (Raleigh, NC, USA) at North Carolina State University and a sample from each experimental diet was sent to the North Carolina Department of Agriculture and Consumer Services for proximate analysis of nutrient composition (Raleigh, NC, USA).

**Table 3.** Composition of basal diets (as-fed basis)

Item	Phase 1	Phase 2	Phase 3
**Ingredient, %**			
**Corn, yellow**	38.45	47.99	64.30
**Whey permeate**	20.00	13.00	0.00
**Soybean meal, 48% CP**	17.00	23.00	28.00
**Poultry meal**	10.00	5.00	0.00
**Fish meal**	5.00	3.00	0.00
**Blood plasma**	3.00	1.50	0.00
**Poultry fat**	1.50	1.30	1.50
**SBM60Corn40^1^**	3.00	3.00	3.00
**L-Lys HCl**	0.41	0.36	0.41
**L-Met**	0.22	0.16	0.14
**L-Thr**	0.19	0.14	0.15
**L-Trp**	0.03	0.00	0.00
**Dicalcium phosphate**	0.00	0.20	1.20
**Limestone**	0.80	0.95	0.90
**Vitamin premix^2^**	0.03	0.03	0.03
**Mineral premix^3^**	0.15	0.15	0.15
**Salt**	0.22	0.22	0.22
**Calculated composition:**			
**Dry matter, %**	92.2	91.1	89.3
**ME, kcal/kg**	3,449	3,401	3,357
**CP, %**	25.3	23.0	20.2
**SID Lys, %**	1.50	1.36	1.23
**SID Met + Cys, %**	0.82	0.74	0.69
**SID Trp, %**	0.27	0.23	0.21
**SID Thr, %**	0.89	0.80	0.73
**SID Val, %**	0.98	0.89	0.78
**Ca, %**	0.95	0.81	0.72
**STTD P, %**	0.50	0.39	0.34
**Total P, %**	0.75	0.63	0.60

1 SBM60Corn40 is a mix of 60% soybean meal, 48% CP, and 40% corn, yellow.

2 The vitamin premix provided the following per kilogram of complete diet: 6,613.8 IU of vitamin A as vitamin A acetate, 992.0 IU of vitamin D3, 19.8 IU of vitamin E, 2.64 mg of vitamin K as menadione sodium bisulfate, 0.03 mg of vitamin B12, 4.63 mg of riboflavin, 18.52 mg of D-pantothenic acid as calcium pantothenate, 24.96 mg of niacin, and 0.07 mg of biotin.

3 The trace mineral premix provided the following per kilogram of complete diet: 4.0 mg of Mn as manganous oxide, 165 mg of Fe as ferrous sulfate, 165 mg of Zn as zinc sulfate, 16.5 mg of Cu as copper sulfate, 0.30 mg of I as ethylenediamine di-hydroiodide, and 0.30 mg of Se as sodium selenite.

SID, standardized ileal digestible; STTD P, standardized total tract digestible phosphorus.

### Experimental procedures and sample collection

Individual BW and feed intake were recorded at the end of each phase to calculate average BW, average daily gain (ADG), average daily feed intake (ADFI), and gain to feed ratio (G: F). The fecal score was recorded once a day by the same individual throughout the entire experimental period, based on a visual 1 to 5 scale: 1) very hard and dry feces, 2) firm stool, 3) normal stool, 4) loose stool, and 5) watery stool with no shape. Fecal scoring began on d 3 of the experimental period due to inconsistencies in how quickly pigs began to eat upon placement.

On day 28 of the study, all pigs were humanely euthanized via exsanguination after incapacitation by a captive bolt gun to the head. Once euthanized, the digestive tract was removed for sample collection. Samples of the mid-jejunum, 3 m after the pyloric duodenal junction, were removed and rinsed with a sterile saline solution (0.9%) to clear the sample of digesta content. A portion of the mid-jejunum sample was cut lengthwise and splayed open to obtain intestinal mucosa samples by scraping with a glass microscope slide. The scraped mucosa was transferred into Eppendorf tubes (2 mL) and were immediately placed into liquid nitrogen and were subsequently stored at –80°C for further analysis of mucosa-associated oxidative stress and immune parameters, as well as relative abundance and diversity of the jejunal mucosa-associated microbiota. An additional segment of the mid-jejunum was removed, rinsed with a sterile saline solution, and transferred to a 5 mL tube, immediately placed into liquid nitrogen, and then stored at –80°C for further analysis of gene expression in jejunal tissue. A final segment of the mid-jejunum was removed, rinsed with a sterile saline solution, and collected in a 50 mL Falcon tube containing 10% buffered formaldehyde and stored for further evaluation of intestinal histology including villus height, crypt depth, and enterocyte proliferation rate.

### Diversity and relative abundance of jejunal mucosa-associated microbiota

Samples of mucosa collected from the mid-jejunum were utilized for analysis of the diversity and relative abundance of the mucosa-associated microbiota. Following the protocol provided by Zymo Research (Irvine, CA, USA), the scraped mucosa samples were prepared and sent to Zymo Research for 16S rRNA microbiome sequencing analysis. Deoxyribonucleic acid (DNA) was extracted from the mucosa samples through use of the ZymoBIOMICS-96 MagBead DNA Kit (Zymo Research). Using the Quick-16S Primer Set V3-V4 (Zymo Research), the previously extracted DNA is prepared for targeted sequencing. The NGS Library Preparation Kit is also utilized for further microbial analysis, and the primers contained by the NGS Library Preparation Kit as specifically designed by Zymo Research to ensure total coverage of the 16S gene. The result of the DNA preparations was then quantified using qPCR fluorescence readings and are subsequently pooled based on molarity. To refine the readings, the pooled readings were entered into the Select-a-Size DNA Clean & Concentrator (Zymo Research), quantified using TapeStation (Agilent Technologies, Santa Clara, CA, USA) and Qubit (Thermo Fisher Scientific Inc., Waltham, MA). Finally, the library was sequenced using Illumina NextSeq 2000, with a p1 (CAT No. 20075294) reagent kit (600 cycles) with a 30% PhiX spike-in utilized during sequencing. To infer unique amplicon sequences from the raw reads, the DADA2 pipeline was utilized (Callahan et al., 2016). To assign taxonomy, an internally curated 16S database, referencing Greengenes and Silva 16S databases, was used, alongside Uclust from QUIIME v.1.9.1. Alpha diversity, including Chao1, Shannon, and Simpson indices, was evaluated using the website program, MicrobiomeAnalyst (QC, CA, USA) as previously described (Deng et al., 2023b). The amplicon sequence variant (ASV) data were transformed to relative abundance (RA) for further statistical analysis, and the ASV data with less than 0.5% RA at each respective level were combined as “others.” To calculate the RA of gram-positive and gram-negative bacterial populations, the sum of the RA of gram-positive and gram-negative phyla were summed, excluding non-assigned sequences deemed as “others,” and a percentage of assigned sequences was achieved.

### Relative mRNA expression of genes in jejunal tissue

Samples of intestinal tissue taken from the mid-jejunum and weighed (50 to 100 mg) and were homogenized in 1 mL of TRIzol reagent (15-596-026, Invitrogen, Waltham, MA, USA) using the Bead Mill 24 homogenizer (Thermo Fisher Scientific Inc.). Samples were homogenized three times at an energy of 4.5 m/s for 30 s and were placed on ice for 1 min between cycles. Once homogenized, the samples were centrifuged at 12,000×*g* at 4°C for 10 min, as previously described (Jang et al., 2024). The supernatant was removed and transferred into a new 1.5 mL centrifuge tube containing 200 µL of chloroform (Thermo Fisher Scientific Inc.) and was gently vortexed for 1 min until well combined. The tubes containing mixed chloroform and supernatant were incubated for 10 min before centrifugation for 15 min 12,000×*g* at 4°C. After centrifugation, the tubes were carefully removed from the centrifuge so as not to disrupt the aqueous phase. The aqueous phase was then removed and transferred to a tube containing 200 µL of isopropanol and was gently vortexed for 1 min until thoroughly combined. The mixed samples were allowed to incubate at room temperature for 10 min before a final centrifugation for 15 min at 12,000×*g* at 4°C. The supernatant obtained from centrifugation was carefully removed to retain the pellet inside the tube. Tubes were opened and placed to dry in a sterilized fume hood for approximately 30 min until all the remaining supernatant had evaporated. The RevertAid First Strand cDNA Synthesis kit (#01299151, Thermo Fisher Scientific Inc.) was used to obtain cDNA from the extracted RNA. All procedures were conducted following the instructions associated with the kit. To conduct quantitative real-time polymerase chain reaction (RT-qPCR), the CFX Connect Real-Time PCR Detection System (BioRad, Hercules, CA, USA) and the Maxima SYBR Green/ROX qPCR Master Mix (#01292815, Thermo Fisher Scientific Inc.) were used. The run conditions included 6 steps where the machine cycled at the following temperatures and hold times, 1) 50°C for 2 min; 2) 95°C for 10 min; 3) 95°C for 15 s; 4) 60°C for 30 s; 5) 72°C for 30 s; 6) plate read. After the conclusion of a full cycle including a plate read, steps 3 through 6 were repeated an additional 39 times before termination of the cycle. The primers used are described in Table 4 and were synthesized by a commercial company (Millipore Sigma, Burlington, MA, USA). Primer specificity was confirmed by melt curve analysis and all runs produced a single peak without evidence of non-specific amplification (Ririe et al., 1997; Zipper et al., 2004). Glyceraldehyde 3-phosphate dehydrogenase (GAPDH) was utilized as the housekeeping gene and delta-delta Ct values were calculated to obtain the relative gene expression of each respective gene target.

**Table 4.** Sequence of primers for immune response and barrier function in the jejunum of nursery pigs fed diets supplemented with different inclusions of a trub, hops, and yeast mixture (THYM)

Gene	Primer	Sequence	Accession number	Size
**GAPDH**	Forward Reverse	TCGGAGTGAACGGATTTGGC TGCCGTGGGGTGGAATCATAC	NM_001206359.1	20
**Toll-like receptor 2**	Forward Reverse	GGGCTGCGTTCATTCATCAG CTGCAGAGGATGGATGGCAA	XM_005653576.3	132
**Toll-like receptor 4**	Forward Reverse	CGTGCAGGTGGTTCCTAACA GGTTTGTCTCAACGGCAACC	NM_001113039.2	326
**NOD1**	Forward Reverse	AACACCGATCCAGTGAGCAG AAATGGTCTCGCCCTCCTTG	NM_001114277.1	230
**NOD2**	Forward Reverse	GTGCCTCCCCTCTAGACTCA ACGAACCAGGAAGCCAAGAG	NM_001105295.1	191
**Cluster of differentiation 14**	Forward Reverse	CCCTGCCAAATAGACGACGA TCGAGCGTCAGTTCCTTGAG	NM_001097445.2	299
**Interferon-γ**	Forward Reverse	GGCCATTCAAAGGAGCATGG AAGCTCATCTCACCGGAATTT	HQ026021.1	119
**Interlukin-1β**	Forward Reverse	GCCCATCATCCTTGAAACGTG GGAGAGCCTTCAGCATGTGT	NM_214055.1	131
**NF-κB**	Forward Reverse	GCTGGAATGAAGCACGGAAC GCAAGTTGCATGGCCTTCTC	NM_001048232.1	236
**mTOR**	Forward Reverse	TCTCTATCAAGTTGCTGGCCG CTAGCGCTGCCTTTCGAGAT	XM_003127584.6	135
**Zonula occludens-1**	Forward Reverse	CAGAGACCAAGAGCCGTCC TGCTTCAAGACATGGTTGGC	XM_003480423.4	105
**Occludin**	Forward Reverse	TCAGGTGCACCCTCCAGATT AGGAGGTGGACTTTCAAGAGG	XP_005672579.1	169
**ASCT2**	Forward Reverse	GCCAGCAAGATTGTGGAGAT GAGCTGGATGAGGTTCCAAA	XM_003355984.4	206
**CAT1**	Forward Reverse	ATCTCTGCCTGAACAATGCCA ATGCAGTCAAAGCCCACGAA	NM_001012613.1	138
**Glucose transporter 2**	Forward Reverse	TAGAGAAGGCAGGGGCGAC CATCCAAGGCAATTTATCCAGTA	NM_001097417.1	110
**Glucose transporter 5**	Forward Reverse	CCCAGGAGCCGGTCAAG TCAGCGTCGCCAAAGCA	EU012359.2	60
**LAT1**	Forward Reverse	TCAAGTGGGGAACCCTGGTA ATGGAGAGGGGCAGATTCCT	NM_001110421.1	256
**Peptide transporter 1**	Forward Reverse	CCCAGGCTTTGCTACCCAC ACCCGATGCACTTGACGA	NM_214347.1	144
**SGLT1**	Forward Reverse	GCTTTGAATGGAATGCTCTGATT GCATCGTCACCACCCCTG	NM_001164021.1	87

GAPDH, glyceraldehyde-3-phosphate dehydrogenase; NOD1, nucleotide binding oligomerization domain containing 1; NF-κB, nuclear factor-κB; mTOR, mammalian target of rapamycin; CAT, cationic amino acid transporter; LAT, L-type amino acid transporter.

### Inflammatory cytokines, immunoglobulins, and oxidative damage products

Samples of the scraped mucosa taken from the mid-jejunum were weighed (approximately 500 mg) and suspended in phosphate-buffered saline (PBS) (1 mL). The scraped mucosa was then homogenized using a tissue homogenizer (Tissuemiser, Thermo Fisher Scientific Inc.), placing the samples on ice between runs of the tissue homogenizer. After homogenization, the samples were transferred to new 2 mL microcentrifuge tubes and were centrifuged at 14,000×*g* for 15 min as previously described (Holanda and Kim, 2021). Once centrifuged, the resulting supernatant was pulled off and was allocated into five separate 0.5 mL microcentrifuge tubes and stored at –80°C for further analysis. The concentration of total protein, malondialdehyde (MDA), protein carbonyl, immunoglobulin G (IgG), immunoglobulin A (IgA), tumor necrosis factor alpha (TNF-α), interleukin 6 (IL-6), and interleukin 8 (IL-8) were measured using commercial kits following the operation manuals provided in each respective kit.

The supernatant from the initial homogenization of the scraped mucosa samples was further diluted (1:60) in PBS to obtain an appropriate range (20 to 2,000 μg/mL) before measuring the total protein concentration of the diluted scraped mucosal samples using the Pierce BCA Protein Assay Kit (#23225, Thermo Fisher Scientific Inc.) as previously described by Holanda et al. (2020). After completion, the absorbance was read at a wavelength of 562 nm and the final concentrations obtained were used to normalize the results of the subsequent measurements for each unique colorimetric assay. The concentration of MDA in mucosa was determined using the OxiSelect TBARS MDA Quantitation Assay Kit (#STA-330, Cell Biolabs, Inc., San Diego, CA, USA) following the procedure previously described by Moita et al. (2021). The working range for the MDA standards was 0 to 125 µM/L and the absorbance was read at a 532 nm. Values were expressed as nmol/mg protein. To measure protein carbonyl, the OxiSelect Protein Carbonyl ELISA Kit (#STA-310, Cell Biolabs, Inc.) was used, following the procedure previously described by Duarte et al. (2019). Each sample was diluted using PBS to obtain a protein concentration of 10 µg/mL prior to measurement. The standards were prepared to fall within the working range of 0.375 to 7.500 nmol/mg protein. All procedures were conducted following the instruction manual provided by the kit manufacturer. The absorbance was measured at a wavelength of 450 nm. The concentration of IgG and IgA were measured using the ELISA kits (E101-104 and E101-102, Bethyl Laboratories, Inc., Montgomery, TX, USA), as described by Cheng et al. (2022). Supernatant from the mucosal samples were diluted with PBS to 1:1,000 for both IgG and IgA, respectively, to achieve the proper working range for measurement. The absorbance was read at a wavelength of 450 nm. The concentration was expressed as µg/mg of protein. The Porcine TNF-α Immunoassay Kit (#PTA00, R&D Systems, Minneapolis, MN, USA) was used to measure the TNF-α concentration as also previously described (Cheng et al., 2022). The absorbance was read at wavelengths of 450 and 570 nm, with 570 nm being used for correction. The concentration of TNF-α obtained was expressed as pg/mg protein. The IL-6 concentration was measured using the Porcine IL-6 DuoSet ELISA kit (#DY686, R&D Systems) following Deng et al. (2023a). The samples were not diluted prior to analysis, following the procedure provided by the kit manufacturer. The absorbance was read at wavelengths of 450 and 570 nm, and the reading from the 570 nm was used to correct the 450 nm reading. The Porcine IL-8/CXCL8 DuoSet ELISA kit (#DY535, R&D Systems) was used to obtain the IL-8 concentration as previously described (Jang and Kim, 2019). The samples were diluted with reagent diluent in a 1:5 ratio prior to analysis. The absorbance was read at wavelengths of 450 and 570 nm, and the reading from the 570 nm was used to correct the 450 nm reading. All procedures were conducted following the protocol provided by the kit manufacturer.

### Intestinal morphology and Ki-67 in crypt cells

After sampling, sections taken from the mid-jejunum were fixed in 10% formalin for 3 d. The samples were then removed from the 10% formalin, cut transversely into two sections, placed into a plastic cassette, and were transferred to a 70% ethanol solution for storage. The cassettes were delivered to the University of North Carolina School of Medicine Lineberger Comprehensive Cancer Center (Chapel Hill, NC, USA) for dehydration, embedment, staining and immunohistochemistry of Ki-67 proteins using their internal protocol as described by Baker et al. (2024b). Processed samples were mounted on slides and were evaluated using an Olympus CX31 microscope (Lumenera Corporation, Ottowa, Canada) and the Infinity 2-2 digital CCD software. For each slide, 10 images were taken from various locations, where the entire length of the villus and the associated crypt were easily visible. The 10 images of each slide were imported into the Teledyne Lumenera Infinity Analyze 7 software to analyze intestinal morphology and determine the number of Ki-67 positive cells. The villus length was considered to be from the top of the villus to the villus-crypt junction, and the crypt depth was considered to be from the villus-crypt junction to the bottom of the crypt. The villus height to crypt depth (VH:CD) ratio was calculated by dividing the measured villus height by the measured crypt depth. The number of Ki-67 positive cells was determined by manual count. The same images were used to calculate the percentage of Ki-67 positive cells from total cells in the crypt. Images were cropped and then imported into the ImageJS software and were reported as a ratio of Ki-67 positive cells to total cells in the crypt (%). All procedures related to analysis of intestinal morphology were conducted by the same person and the 10 measurements per pig were averaged and reported as a single value per pig (Cheng et al., 2021; Supplementary Figure S1, see online supplementary material for a color version of this figure).

### Statistical analysis

An initial power test was conducted to determine the number of replications required to detect a statistically significant mean difference of 13 to 14% at *P *< 0.05. Based on previous studies at the same research facility using pigs of a similar genetic background (Moita and Kim, 2023; Gormley et al. 2024), a coefficient of variation of 7.5% was assumed. The power of test (1—beta) was set at 0.80 with a 95% confidence level, resulting in a minimum of 8 replications per treatment (Martin et al., 1987). A two-tailed t-test was used to test the effects of heat treatment on the percentage of lysed yeast cells in samples dried with and without heat treatment. Data from the animal trial were analyzed using the MIXED procedure in SAS 9.4 (SAS Inc., Cary, NC, USA). The dietary treatment, the main effect, was considered the fixed effect, and the initial BW and sex blocks were considered random effects. Preplanned orthogonal polynomial contrasts were used to determine the linear or quadratic effects of increasing levels of THYM. The individually housed pig served as the experimental unit for all analyses. Results were considered statistically significant when the *P* value was less than 0.05 and were considered a tendency when the *P* value was between 0.05 and 0.10.

## Results

### Diversity and relative abundance of jejunal mucosa-associated microbiota

At the genus level, no differences in α-diversity of the jejunal mucosa-associated microbiota were observed with increasing inclusion of THYM in the diet (Table 5). At the phylum level, the relative abundance of the jejunal mucosa-associated microbiota was not affected by increasing inclusion of THYM in the diet. At the family level, the relative abundance of the jejunal mucosa-associated microbiota was not affected with the increasing inclusion of THYM in the diet (Table 6). At the genus level, the relative abundance of *Sharpea* tended to linearly increase (*P = *0.079) with increasing inclusion of THYM in the diet. The other genera were not affected by the increasing inclusion of THYM. At the species level, the relative abundance of *Limosilactobacillus mucosae* linearly decreased (*P < *0.05) with the increasing inclusion of THYM in the diet. In addition, there was a tendency for the relative abundance of *Sharpea azabuensis* to linearly increase (*P = *0.079) with the increasing inclusion of THYM in the diet.

**Table 5.** Alpha diversity at the genus level and relative abundance at the phylum level, of mucosa-associated microbiota in the jejunum, of nursery pigs fed diets supplemented with different inclusions of a trub, hops, and yeast mixture (THYM)^1^

	THYM, %				SEM	*P* value	
Item	0.0	0.7	1.4	2.1		Linear	Quadratic
**Alpha diversity, genus**							
**Chao1**	42.8	47.3	41.8	40.0	3.9	0.432	0.425
**Shannon**	1.9	1.9	1.9	1.9	0.3	0.859	0.892
**Simpson**	0.72	0.70	0.68	0.71	0.06	0.842	0.615
**Relative abundance, phylum, %**							
**Gram-positive**	74.0	83.8	80.1	79.8	9.0	0.678	0.502
**Bacillota**	48.7	58.4	45.0	50.8	6.1	0.436	0.478
**Actinomycetota**	24.5	24.7	33.5	33.9	6.7	0.205	0.991
**Gram-negative**	26.0	16.2	19.9	20.2	9.0	0.678	0.502
**Pseudomonadota**	18.7	10.5	14.3	13.1	7.6	0.668	0.611
**Bacteriodota**	7.0	5.6	4.7	5.7	2.7	0.699	0.667
**Others**	1.2	0.8	2.5	1.4	1.0	0.523	0.643

1 Each least squares mean represents eight observations.

**Table 6.** Relative abundance of mucosa-associated microbiota in the jejunum, at the family, genus, and species level, of nursery pigs fed diets supplemented with different inclusions of a trub, hops, and yeast mixture (THYM)^1^

	THYM, %				SEM	*P* value	
Item	0.0	0.7	1.4	2.1		Linear	Quadratic
**Family**							
**Lactobacillaceae**	26.8	33.8	21.9	24.4	7.0	0.527	0.741
**Bifidobacteriaceae**	20.3	20.6	30.3	29.5	6.7	0.196	0.928
**Helicobacteraceae**	17.8	9.8	13.8	11.8	7.4	0.647	0.666
**Veillonellaceae**	8.1	10.9	6.4	6.1	2.1	0.272	0.481
**Prevotellaceae**	6.5	4.8	4.5	5.2	2.5	0.702	0.640
**Lachnospiraceae**	5.2	3.6	4.4	5.0	1.5	0.950	0.489
**Coriobacteriaceae**	4.1	4.1	3.1	4.3	1.1	0.950	0.575
**Ruminococcaceae**	3.2	2.4	3.0	2.9	1.6	0.974	0.813
**Erysipelotrichaceae**	1.3	1.4	1.3	2.5	0.6	0.142	0.323
**Streptococcaceae**	0.7	1.1	0.7	0.8	0.3	0.974	0.505
**Succinivibrionaceae**	0.4	0.4	0.4	0.7	0.4	0.508	0.622
**Peptostreptococcaceae**	0.4	0.7	0.6	0.5	0.4	1.000	0.591
**Acidaminococcaceae**	0.5	0.4	0.4	0.4	0.2	0.806	0.811
**Others**	4.9	6.1	9.3	5.9	3.5	0.693	0.518
**Genus**							
***Lactobacillus***	26.8	33.8	21.9	24.4	7.0	0.527	0.741
***Bifidobacterium***	20.3	20.6	30.3	29.5	6.7	0.196	0.928
***Helicobacter***	17.8	9.8	13.8	11.8	7.4	0.647	0.666
***Olsenella***	3.8	3.7	2.7	4.0	1.1	0.937	0.515
***Prevotella***	2.9	2.5	1.6	3.1	1.4	0.964	0.502
***Mitsuokella***	3.0	3.9	2.2	2.1	1.0	0.308	0.616
***Megasphaera***	1.9	3.6	1.7	1.8	0.6	0.423	0.171
***Dialister***	1.4	1.6	1.0	1.2	0.4	0.471	0.978
***Selenomonas***	0.6	1.2	0.6	0.4	0.6	0.641	0.529
***Syntrophococcus***	1.1	0.8	1.0	2.1	0.5	0.125	0.142
***Blautia***	1.2	0.7	0.8	0.8	0.4	0.523	0.590
***Streptococcus***	0.7	1.1	0.7	0.8	0.3	0.974	0.505
***Roseburia***	0.6	0.6	0.4	0.2	0.3	0.399	0.716
***Sharpea***	0.2	0.6	0.3	1.1	0.3	0.079	0.442
***Succinivibrio***	0.3	0.2	0.3	0.7	0.4	0.333	0.498
**Others**	17.4	15.4	20.6	16.1	7.0	0.972	0.860
**Species**							
***Bifidobacterium thermacidophilum-thermophilum***	16.2	17.9	27.3	26.9	6.3	0.121	0.863
***Lactobacillus delbrueckii***	12.8	29.9	19.2	18.4	6.1	0.812	0.121
***Helicobacter rappini***	17.8	9.8	13.8	11.8	7.3	0.651	0.669
***Bifidobacterium boum***	4.0	2.5	2.6	1.7	1.2	0.198	0.827
***Olsenella profusa***	2.3	2.9	2.1	3.1	0.8	0.661	0.758
***Prevotella copri***	2.3	1.6	1.3	2.5	1.2	0.953	0.439
***Mitsuokella multacida***	2.5	2.9	1.7	1.7	0.9	0.362	0.771
***Limosilactobacillus mucosae***	5.3	2.9	1.0	0.6	1.4	0.005	0.412
***Dialister succinatiphilus***	0.8	1.5	0.6	1.0	0.4	0.813	0.704
***Olsenella umbonata***	1.3	0.5	0.5	0.7	0.6	0.420	0.358
***Selenomonas bovis***	0.6	1.2	0.6	0.4	0.6	0.641	0.529
***Sharpea azabuensis***	0.2	0.6	0.3	1.1	0.3	0.079	0.442
**Others**	33.8	25.8	29.0	30.2	8.8	0.847	0.605

1 Each least squares mean represents eight observations.

### Relative mRNA expression of genes in jejunal tissue

In jejunal tissue, the relative gene expression of nucleotide binding oligomerization domain containing (NOD) 2 and interferon (IFN)-γ were linearly increased (*P *< 0.05) with increasing inclusion of THYM in the diet (Table 7). The relative gene expression of peptide transporter (PEPT) 1 and sodium-glucose cotransporter (SGLT) 1 were linearly decreased (*P *< 0.05) with increasing inclusion of THYM in the diet. The relative gene expression of Toll-like receptor (TLR) 2 tended to be linearly increased (*P = *0.058) with increasing inclusion of THYM in the diet. In contrast, the relative gene expression of the alanine, serine, cysteine transporter (ASCT) 2 tended to be linearly decreased (*P *= 0.093) with increasing inclusion of THYM in the diet. The relative gene expression of zonula occludens-1 (ZO-1) and occludin exhibited a quadratic response (*P *< 0.05) with increasing inclusion of THYM in the diet. Finally, the relative gene expression of ASCT2 tended to exhibit a quadratic response (*P *= 0.082) with increasing inclusion of THYM in the diet.

**Table 7.** Relative gene expression in jejunal tissue of nursery pigs fed diets supplemented with different inclusions of a trub, hops, and yeast ­mixture (THYM)^1^

	THYM, %				SEM	*P* value	
Item	0.0	0.7	1.4	2.1		Linear	Quadratic
**Toll-like receptor 2**	1.00	1.51	2.16	1.91	0.38	0.058	0.328
**Toll-like receptor 4**	1.00	0.94	1.38	0.91	0.36	0.798	0.569
**NOD1**	1.00	0.94	1.10	1.31	0.23	0.314	0.582
**NOD2**	1.00	1.48	1.61	1.66	0.23	0.043	0.350
**Cluster of differentiation 14**	1.00	1.00	1.09	1.09	0.24	0.749	0.999
**Interferon-γ**	1.00	1.35	1.70	2.77	0.39	0.003	0.259
**Interlukin-1β**	1.00	1.33	1.19	1.38	0.22	0.310	0.768
**NF-κB**	1.00	1.09	1.31	1.14	0.14	0.301	0.341
**Mtor**	1.00	0.98	0.77	0.83	0.19	0.313	0.798
**Zonula occludens-1**	1.00	1.40	1.46	1.12	0.20	0.545	0.033
**Occludin**	1.00	2.72	2.09	1.20	0.36	0.983	0.001
**ASCT2**	1.00	0.62	0.56	0.68	0.15	0.093	0.082
**CAT1**	1.00	0.58	0.81	0.71	0.15	0.362	0.309
**Glucose transporter 2**	1.00	1.62	1.30	1.30	0.33	0.673	0.312
**Glucose transporter 5**	1.00	0.95	0.71	0.73	0.14	0.103	0.798
**LAT1**	1.00	0.67	0.74	0.79	0.20	0.541	0.350
**Peptide transporter 1**	1.00	1.22	0.68	0.50	0.21	0.034	0.333
**SGLT1**	1.00	1.05	0.46	0.41	0.32	0.013	0.788

1 Each least squares mean represents eight observations.

NOD1, nucleotide binding oligomerization domain containing 1; NF-κB, nuclear factor-kappa B; CAT, cationic amino acid transporter; LAT, L-type amino acid transporter.

### Oxidative stress status, humoral immune status, intestinal inflammatory status and intestinal morphology, and crypt cell proliferation

In the jejunal mucosa, there were no differences observed related to oxidative stress status, humoral immune status, or intestinal inflammatory status, with increasing inclusion of THYM in the diets (Table 8). Villus height, crypt depth, VH:CD, and Ki-67 positive cell count in the crypt were not affected by the increasing inclusion of THYM in the diets. However, the percentage of Ki-67 positive cells in the crypt was linearly increased (*P < *0.05) with increasing inclusion of THYM in the diets.

**Table 8.** Products of oxidative damage and immune response, and intestinal morphology of jejunal tissue, of nursery pigs fed diets supplemented with different inclusions of a trub, hops, and yeast mixture (THYM)^1^

	THYM, %				SEM	*P* value	
Item	0.0	0.7	1.4	2.1		Linear	Quadratic
**Unit/mg protein**							
**TNF-α, pg**	1.51	1.30	2.17	2.13	0.54	0.199	0.852
**IL-6, pg**	10.88	10.40	17.71	12.92	3.24	0.351	0.507
**IL-8, pg**	0.25	0.20	0.21	0.29	0.04	0.567	0.138
**IgA, µg**	2.68	2.87	2.51	2.63	0.66	0.864	0.958
**IgG, µg**	1.84	1.59	1.89	2.00	0.18	0.328	0.312
**MDA, nmol**	0.21	0.12	0.36	0.23	0.09	0.410	0.801
**Protein carbonyl, nmol**	1.36	1.07	1.20	1.29	0.33	0.943	0.568
**Intestinal morphology**							
**Villus height, µm**	495	474	485	474	36	0.713	0.878
**Crypt depth, µm**	194	173	185	195	10	0.738	0.156
**VH:CD**	2.6	2.8	2.7	2.4	0.2	0.488	0.222
**Ki-67^+^,^2^ count**	43.0	44.4	45.2	45.4	2.3	0.324	0.713
**Ki-67^+^,^3^ %**	34.7	40.3	42.1	42.5	2.2	0.011	0.214
**Villus height, µm**	495	474	485	474	36	0.713	0.878

1 Each least squares mean represents eight observations.

2 Number of Ki-67 positive cells in the crypt.

3 Number of Ki-67 positive cells to total cells in the crypt, as a percentage.

### Growth performance and fecal score

The BW, ADG, and ADFI of pigs were not affected by increasing inclusion of THYM in the diets (Table 9). There were no differences in G:F in phase 1 or phase 2; however, G:F in phase 3 and the overall experimental period was linearly increased (*P *< 0.05) with increasing inclusion of THYM in the diets. During the entire experimental period, there were no differences in fecal score with increasing inclusion of THYM in the diets.

**Table 9.** Growth performance and fecal score of nursery pigs fed diets supplemented with different inclusions of a trub, hops, and yeast mixture (THYM)^1^

	THYM, %				SEM	*P* value	
Item	0.0	0.7	1.4	2.1		Linear	Quadratic
**BW, kg**							
**Day 0**	6.8	6.8	6.8	6.8	0.3	0.953	0.937
**Day 9**	7.2	7.0	7.3	7.1	0.5	0.908	0.982
**Day 20**	11.2	11.2	11.3	11.5	0.9	0.629	0.753
**Day 28**	16.3	16.0	16.5	17.0	1.0	0.382	0.516
**ADG, g/d**							
**Day 0 to 9 (P1)**	45	27	55	38	24	0.929	0.981
**Day 9 to 20 (P2)**	370	383	365	403	44	0.570	0.677
**Day 20 to 28 (P3)**	726	691	739	775	31	0.164	0.261
**Day 0 to 28 (Overall)**	341	332	346	364	30	0.378	0.501
**ADFI** ^2^ **, g/d**							
**Day 0 to 9 (P1)**	121	97	99	97	17	0.141	0.287
**Day 9 to 20 (P2)**	493	492	437	478	51	0.628	0.641
**Day 20 to 28 (P3)**	985	959	967	979	47	0.963	0.683
**Day 0 to 28 (Overall)**	480	464	446	464	32	0.620	0.563
**G:F^3^**							
**Day 0 to 9 (P1)**	0.38	-0.12	0.49	0.37	0.28	0.562	0.427
**Day 9 to 20 (P2)**	0.80	0.80	0.84	0.86	0.04	0.156	0.756
**Day 20 to 28 (P3)**	0.74	0.72	0.76	0.81	0.03	0.032	0.192
**Day 0 to 28 (Overall)**	0.74	0.72	0.81	0.80	0.02	0.011	0.769
**Fecal score**							
**Day 3 to 9 (P1)**	3.4	3.2	3.4	3.5	0.1	0.467	0.345
**Day 9 to 20 (P2)**	3.3	3.3	3.3	3.2	0.1	0.593	0.893
**Day 20 to 28 (P3)**	3.1	3.1	3.1	3.1	0.1	0.863	0.782
**Day 3 to 28 (Overall)**	3.3	3.2	3.3	3.3	0.1	0.753	0.556

1 Each least squares mean represents eight observations.

2 Data from a single pig from the 0.0 treatment were excluded from analysis due to excessive feed wastage into the pit that could not be recovered for measurement.

3 Data from a single pig from the 0.0 treatment were excluded from analysis due to excessive feed wastage into the pit that could not be recovered for measurement.

## Discussion

Craft breweries generate a large volume of co-products that often burden local infrastructure, as most co-products are disposed of as sewage (Garcia-Garcia et al., 2017). Meanwhile, nursery diets are the most expensive of all phases of pig feeding, placing economic pressure on producers (Skinner, 2014). Notably, spent yeast from craft brewing has approximately 5 times the content of hop acids as spent yeast from multinational breweries (Bryant and Cohen, 2015) and therefore could provide additional benefits compared to co-products derived from multinational breweries. The combined use of a trub, hops and yeast mixture derived as a co-product of the craft brewing process, may support intestinal development and reduce the harmful effects of weaning on intestinal physiology, when fed to nursery pigs (Kim and Hansen, 2022). Utilizing these co-products in nursery pig diets also aligns with the goals of the animal agriculture industry related to sustainability, offering benefits to both the craft brewing and animal agriculture industries.

Just as the jejunum is a key site for assessing intestinal health because of its role in nutrient digestion and absorption, the jejunal mucosa-associated microbiota warrants investigation due to its unique composition and direct interactions with the intestinal epithelial cells. The populations of the mucosa-associated microbiota greatly differ from those found within the luminal microbiota (Adhikari et al., 2019), therefore, changes of the relative abundance of members of the mucosa-associated microbiota can have significant implications related to host immune system activation (Daniel et al., 2021). Increasing inclusion of THYM decreased the relative abundance of *Limosilactobacillus mucosae.* A previous study found that this species can mitigate the negative effects of dysbiosis by reducing intestinal inflammation and protecting against mucosal damage, thereby reducing diarrhea (Li et al., 2023). In this study, however, the reduction of *Limosilactobacillus mucosae* did not correspond with changes in intestinal inflammation or fecal score, likely because the relative abundance of the rest of the mucosa-associated microbiota remained stable. The absence of changes in alpha diversity further supports the conclusion that THYM did not exacerbate any weaning-related shifts to the relative abundance of the mucosa-associated microbiota and therefore the protective role of *Limosilactobacillus mucosae* was not required. It is important to note that interpretation of genus-level shifts in the mucosa-associated microbiota is inherently limited, as minor changes to the overall population do not necessarily reflect functional outcomes. Nevertheless, these findings can provide ecological context to understand the potential microbial contributions to host responses observed in this study. Although hop acids and metabolites are known to have antimicrobial properties (Bartmańska et al., 2018), these effects were not observed in the jejunal mucosa-associated microbiota in this study. It is possible that the antimicrobial effects of THYM would be more pronounced in pigs challenged with an enteric pathogen, as the pigs in this study were generally healthy.

The increasing dietary inclusion of THYM in the diets of nursery pigs influenced the expression of genes related to the innate immune response. Namely, it was found that the relative gene expression of TLR2, NOD2, and IFN-γ increased with increasing dietary inclusion of THYM. The simultaneous increase in the expression of these genes could indicate a positive relationship between the functional components of THYM and innate immune system recognition and signaling. The expression of TLR2 is typically associated with the sensing of microbial-associated molecular patterns (MAMP) in the cell wall of gram-positive bacteria, such as lipoteichoic acid and peptidoglycan (Mogensen, 2009), but can also sense MAMP derived from other sources, like viral glycoproteins or fungal glucans (Oliveira-Nascimento et al., 2012). The observed increase in TLR2 could be indicative of immune activation related to the β-D-glucans and mannan oligosaccharides of the yeast cell wall fragments found within THYM (Brown and Gordon, 2003; Davis et al., 2004). In addition to TLR2, NOD2 is pattern recognition receptor (PRR) that is classically known to detect constituents of bacterial peptidoglycan (Inohara et al., 2003), whereas NOD2 also plays a role in the recognition of chitin, a common component of the yeast cell wall (Wagener et al., 2014; Chang et al., 2025). Considering the minimal changes in the jejunal mucosa-associated microbiota, it is likely that the contribution of yeast cell wall components from THYM played an active role in increasing the gene expression of these PRR. Different than TLR2 and NOD2, IFN-γ is a cytokine responsible for a wide array of immune functions, primarily related to activation of type 1 immunity, which is associated with cell-mediated defense against viral and bacterial infections (Schroder et al., 2004). The role of yeast cell wall components has been investigated and similar effects on the innate immune system were observed in previous studies (Shen et al., 2009; Lee et al., 2021; Duarte and Kim, 2024; Gormley et al., 2024).

In addition to the changes in the expression of genes related to the innate immune system, a decrease in the relative gene expression of active nutrient transporters, PEPT1, SGLT1, and ASCT2, was observed with the increasing dietary inclusion of THYM. Decreased relative gene expression of nutrient transporters has been proposed as an adaptive response when nutrient availability is sufficient, potentially reducing the energetic cost of maintaining a high absorptive capacity (Vigors et al., 2016; Yang et al., 2016). Such a shift could modestly contribute to reduced energy expenditure at the level of jejunal tissue. The inclusion of THYM also had a quadratic effect on the gene expression of tight junction proteins, ZO-1 and occludin, key structural components related to intestinal barrier integrity (Hu et al., 2013). At moderate inclusion levels, THYM may have enhanced intestinal repair through upregulation of these barrier-associated genes. At higher inclusion rates, however, the increased rate of intestinal cell turnover could have limited functional enterocyte maturity, contributing to the observed quadratic response.

In this study there were no observed differences in the intestinal morphology parameters of villus height, crypt depth, and VH:CD, which can serve as indicators of absorptive potential, nutrient digestibility, and growth performance (Adeola and King, 2006; Tsukahara et al., 2013; Chen et al., 2020). There was, however, an increase in the percentage of Ki-67 positive cells in the crypt of the jejunum, with increasing inclusion of THYM in the diets. The percentage of Ki-67 positive cells differs from the Ki-67 count, in that the crypt depth can influence the total cell count in the jejunal crypt (Hampson, 1986). The increasing percentage of Ki-67 proliferative to non-proliferative cells in the crypt could indicate enhanced intestinal cell turnover and could potentially be related to the increase in the relative gene expression of IFN-γ. Although more commonly associated with its role as an inflammatory cytokine, IFN-γ also is active in the regulation of cellular proliferation (Asao and Fu, 2000). Increased intestinal cell turnover has been shown to be related to improved intestinal repair from the damages after weaning and thus finally related to improved efficiency of nutrient absorption and utilization (Willing and Van Kessel, 2007; Kim et al., 2019). Additionally, IFN-γ can enhance nutrient delivery by stimulating nitric oxide production, inducing vasodilation and improving blood flow (Alican and Kubes, 1996; Lin et al., 2008). Together, this suggests that the reduced gene expression of the active nutrient transporters could be due to the increased efficiency of nutrient absorption as influenced by IFN-γ.

Collectively, these findings provide a potential mechanism to explain the observed increase in G:F with increasing THYM inclusion, in the overall experimental period. The G:F is a key metric in pig production, both from an economic and sustainability perspective (Patience et al., 2015), as feed costs represent a large proportion of production expenses (Niemi et al., 2010). The combined genus-level shifts in the mucosa-associated microbiota and bioactive components within THYM may have contributed to the observed changes in relative gene expression observed in this study (Duarte and Kim, 2022). Decreased activation of genes related to innate immune system activation are consistent with a decreased allocation of resources away from nutrient digestion and absorption, thereby improving growth performance (Duarte et al., 2020; Xu et al., 2022). Additionally, the increased crypt cell proliferation in the absence of inflammation could indicate an accelerated rate of recovery of the absorptive surfaces after weaning stress (Duarte and Kim, 2024) and thereby supports a more efficient use of dietary nutrients, all of which contributes to the observed G:F response. The inclusion of THYM as a co-product of the craft brewing process could therefore reduce both feed costs and improve sustainability, acting as an alternative feedstuffs that supports improved G:F (Woyengo et al., 2014). In conclusion, the increasing inclusion of a trub, hops, and yeast mixture derived as a co-product from the craft brewing process in the diets of nursery pigs increased the rate of intestinal cell turnover, that maybe have potentially contributed to improvements in feed efficiency. Furthermore, THYM supplementation increased the expression of some genes related to innate immune system recognition and signaling.

## Supplementary Material

skaf425_Supplementary_Data

## Abbreviations:

ADFI: average daily feed intake
ADG: average daily gain
ASCT: alanine, serine, cysteine transporter
BP: breakpoint
BW: body weight
CAT: cationic amino acid transporter
CD: crypt depth
CP: crude protein
DM: dry matter
GAPDH: glyceraldehyde-3-phosphate dehydrogenase
G:F: gain to feed ratio
IFN- γ: interferon γ
IgA: immunoglobulin A
IgG: immunoglobulin G
IL-6: interleukin-6
IL-8: interleukin-8
LAT: L-type amino acid transporter
MAMP: microbial-associated molecular pattern
MDA: malondialdehyde
ME: metabolizable energy
mTOR: mammalian target of rapamycin
NF-kB: nuclear factor-κB
NOD1: nucleotide binding oligomerization domain containing 1
NOD2: nucleotide binding oligomerization domain containing 2
PC: protein carbonyl
PRR: pattern recognition receptor
SGLT: sodium-glucose cotransporter
SID: standardized ileal digestible
STTD: standardized total tract digestible
THYM: trub, hops, and yeast mixture
TLR2: toll-like receptor 2
TLR4: toll-like receptor 4
TNF-α: tumor necrosis factor alpha
VH: villus height
VH:CD: villus height to crypt depth ratio
ZO-1: zonula occludens-1
