# Supplementary material for: Effect of a trub, hops, and yeast mixture on jejunal mucosal immune status, oxidative stress status, structure, and growth performance of nursery pigs
Source: J Anim Sci. 2025 Dec 10;103:skaf425. doi: 10.1093/jas/skaf425 (PMC12759022; doi:10.1093/jas/skaf425)
Supplement: skaf425_Supplementary_Data [file skaf425_supplementary_data.docx]

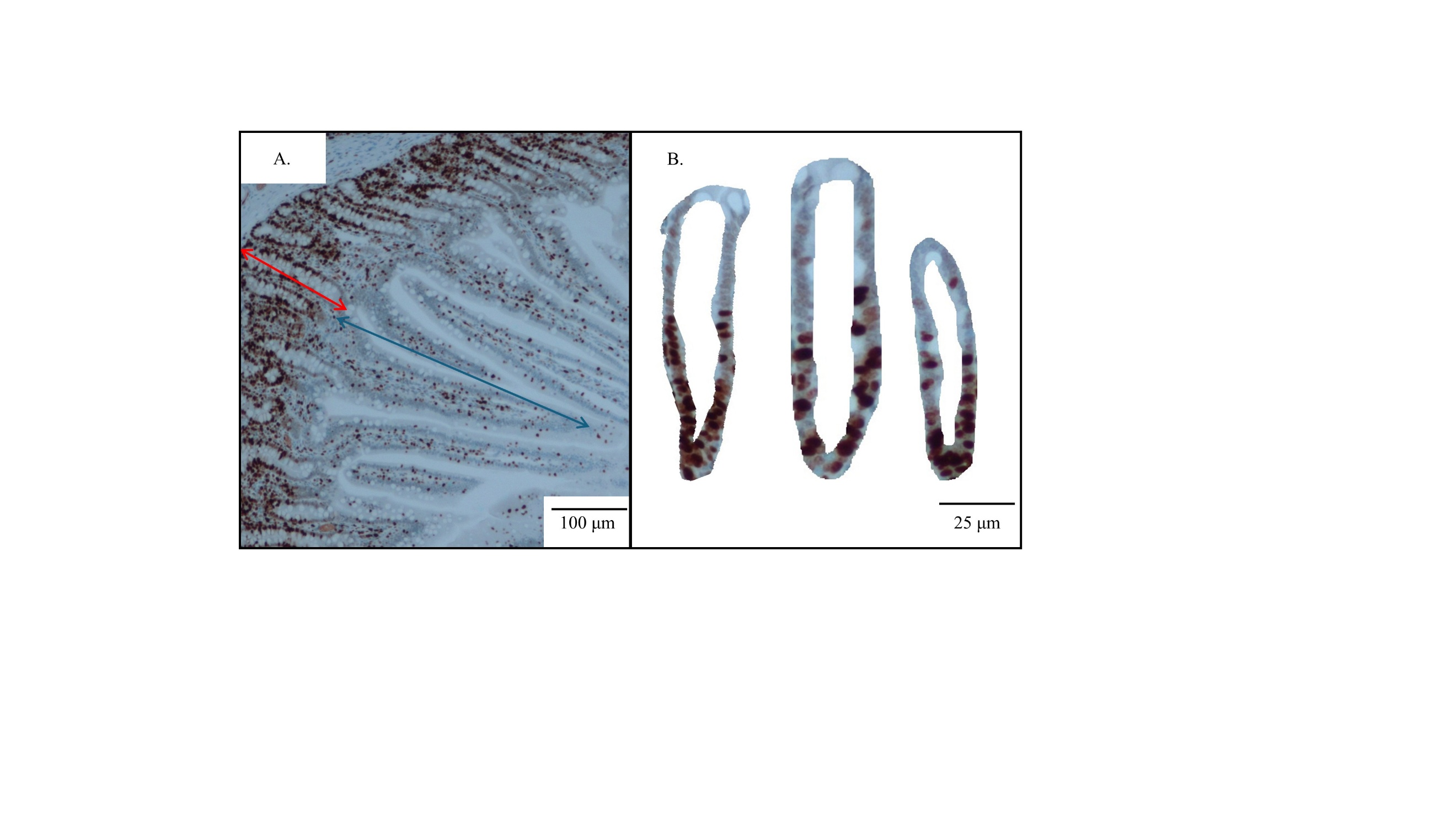


Supplemental Figure 1. Representative images for determination of intestinal morphology and proliferation of cells within the jejunal crypt. Images were taken from mounted slides after immunohistochemistry (Ki-67) staining. Ten images at 40× magnification with clearly visible and well-oriented villi and the associated crypts (A) were obtained to measure villus height (indicated with the double arrow blue line) and crypt depth (indicated with the double arrow red line). Ten images at 100× magnification of the crypts (B) were captured and cropped for manual counting and calculation of Ki-67 proliferation, as a percentage, using the software ImageJS, as indicators of crypt cell proliferation.
